# Optimising the design of internal pilot work to inform efficient randomised controlled trials: issues to consider when developing progression criteria

**DOI:** 10.1186/1745-6215-16-S2-P10

**Published:** 2015-11-16

**Authors:** Kerry Avery, Paula Williamson, Carrol Gamble, Elaine O'Connell Francischetto, Chris Metcalfe, Peter Davidson, Jane Blazeby

**Affiliations:** 1 University of Bristol, Bristol, UK; 2 University of Liverpool, Liverpool, UK; 3 University of Southampton, Southampton, UK

## Background

An internal pilot study is the first part of a substantive RCT where trial parameters are examined and data contribute to the final analyses. This two-phase design provides an opportunity to review pre-agreed ‘progression criteria’ that determine whether the internal pilot should continue into the main trial. Selecting appropriate criteria is critical but little is known about this area. Key issues in selecting progression criteria when designing RCTs with an internal pilot phase are considered.

## Methods

Opinions of stakeholders (funding representatives, methodologists, statisticians, clinicians, trialists) were elicited via a workshop to facilitate discussion regarding optimal use of internal pilots to inform efficient RCTs. This was informed by a literature review of pilot work.

## Results

Three common progression criteria to be considered in pragmatic RCTs with an internal pilot were considered: (i) recruitment; (ii) protocol adherence; (iii) completeness and quality of outcome data. Pre-agreed progression criteria provide an opportunity to review the viability of completing the main trial within the planned timetable and budget, and should be considered alongside other indicators of feasibility rather than simply whether absolute targets were met. Open dialogue between the funder, trial team and steering committee is desirable to address emerging issues and optimally inform decisions about the main trial. This talk will be illustrated with examples, including from surgical trials.

## Conclusion

Careful selection of progression criteria may inform optimal design of RCTs with an internal pilot phase. Flexibility when reviewing progression criteria is necessary when considering the viability of proceeding to the main trial.

